# Moderate- versus high-activity radioactive iodine in intermediate-risk papillary thyroid cancer: a Canadian cohort study

**DOI:** 10.1210/jendso/bvag066

**Published:** 2026-03-24

**Authors:** Mohammad Jay, Iliana C Lega, Noemie Villemure-Poliquin, Cristina Goens, Afshan Zahedi

**Affiliations:** Department of Medicine, University of Toronto, Toronto, Ontario, Canada; Institute of Health Policy, Management and Evaluation, University of Toronto, Toronto, Ontario, Canada; Department of Medicine, University of Toronto, Toronto, Ontario, Canada; Institute of Health Policy, Management and Evaluation, University of Toronto, Toronto, Ontario, Canada; Department of Medicine, Women's College Hospital, Toronto, Ontario, Canada; Institute of Health Policy, Management and Evaluation, University of Toronto, Toronto, Ontario, Canada; Department of Medicine, University of Toronto, Toronto, Ontario, Canada; Department of Medicine, University of Toronto, Toronto, Ontario, Canada; Department of Medicine, Women's College Hospital, Toronto, Ontario, Canada

**Keywords:** papillary thyroid cancer, radioactive iodine therapy, radioactive iodine dose, disease recurrence, prognostic factors, recurrence-free survival

## Abstract

**Context:**

The optimal radioactive iodine (RAI) activity for intermediate-risk papillary thyroid cancer (PTC) remains uncertain, and evidence to guide individualized treatment is limited.

**Objective:**

To compare recurrence between moderate- and high-activity RAI and to identify clinicopathologic factors associated with persistent or recurrent disease.

**Methods:**

We conducted a retrospective cohort study of adults with intermediate-risk PTC treated at a tertiary academic thyroid cancer clinic between 2010 and 2022. Patients received moderate-activity RAI, defined as 30 to 90 mCi, or high-activity RAI, defined as greater than 90 mCi, as initial postoperative therapy. The primary outcome was time to recurrence. The secondary outcome was a composite of persistent or recurrent disease at last follow-up.

**Results:**

Among 181 patients, 94 received moderate-activity RAI and 87 received high-activity RAI. Over a median follow-up of 52 months, 18 recurrences occurred (crude: 3/94 vs 15/87). High-activity RAI was not associated with improved recurrence-free survival (inverse probability of treatment weighting [IPTW]-weighted hazard ratio 2.73; 95% confidence interval [CI] 0.74 to 10.01). Persistent or recurrent disease occurred in 61 patients (34%) with no association in IPTW models (hazard ratio 0.97; 95% CI 0.53 to 1.76). Extranodal extension, microscopic extrathyroidal extension, larger lymph node deposit size, and older age were associated with persistent or recurrent disease.

**Conclusion:**

High-activity RAI was not associated with improved recurrence versus moderate-activity in intermediate-risk PTC. This study is among the first to evaluate moderate-activity RAI as a distinct comparator. Findings support moderate-activity RAI as a reasonable risk-adapted approach. Prospective studies are needed to validate these results.

Papillary thyroid cancer (PTC) is the most common endocrine malignancy, and its incidence continues to rise in high-income countries [1, 2]. Although disease-specific survival exceeds 98%, recurrence occurs in up to 30% of patients with intermediate-risk disease, and optimal postsurgical management remains uncertain [3-7].

Radioactive iodine (RAI) is commonly administered after surgery in intermediate-risk PTC to ablate residual thyroid tissue, treat occult iodine-avid disease, or assess for residual or recurrent disease [8]. However, the optimal therapeutic activity (ie, dose) of RAI remains unclear [9]. Higher activities may improve ablation or target microscopic residual disease but are associated with increased toxicity [8, 10-12]. Therefore, moderate-activity RAI (30-90 mCi) is increasingly favored in practice, but few studies have directly compared oncologic outcomes between moderate and high activities, and no Canadian data exist [8, 10, 11, 13, 14].

Intermediate-risk PTC is a heterogeneous category, and clinicopathologic predictors of recurrence, such as lymph node burden, extranodal extension (ENE), and microscopic extrathyroidal extension (mETE), have demonstrated inconsistent associations with outcomes across studies [15-17]. Better characterization of these features may help guide individualized treatment and surveillance strategies.

The primary objective of this study was to evaluate the association between moderate- vs high-activity RAI and recurrence risk in intermediate-risk PTC. Secondary objectives were to (1) examine a composite outcome of persistent or recurrent disease across moderate- vs high-activity RAI groups, and (2) identify clinicopathologic factors associated with this composite outcome.

## Materials and methods

### Design and setting

We conducted a retrospective cohort study using clinical, pathological, biochemical, and treatment data from a prospectively updated thyroid cancer clinic database used for longitudinal follow-up at the adult Thyroid Cancer Clinic, Women's College Hospital (WCH), a tertiary academic center in Toronto, Canada. The database includes consecutive adults with PTC and is prospectively updated at routine clinical follow-up visits. Baseline clinicopathologic and treatment details were abstracted retrospectively from referral documentation, operative reports, pathology reports, and the institutional nuclear medicine database, as many patients underwent initial management outside our center. The study was approved by the WCH Research Ethics Board and conducted in accordance with institutional and national ethical standards.

### Population

We included patients with intermediate-risk PTC who were assessed between 2010 and 2022 and received either moderate- (1.11-3.33 GBq [30-90 mCi]) or high-activity RAI (>3.33 GBq [>90 mCi]), identified consecutively from the WCH thyroid cancer database. Recurrence risk status was assigned algorithmically using prespecified clinicopathologic variables according to a modified version of the 2015 American Thyroid Association (ATA) recurrence-risk criteria [18]. Detailed operational definitions for risk classification are provided in the Supplementary Methods [19]. Only patients meeting intermediate-risk criteria were included; those with low- or high-risk disease were excluded. Within the intermediate-risk group, we additionally excluded individuals who received no RAI or low-activity RAI (<1.11 GBq [<30 mCi]), as well as patients with missing age or diagnosis date, insufficient outcome data, or a diagnosis date occurring after last follow-up or recurrence. No patients received external-beam radiotherapy, chemotherapy, or tyrosine kinase inhibitor therapy.

### RAI preparation and activity classification

Administered RAI activity was obtained from the institutional nuclear medicine database, which records the delivered (decay-corrected) dose rather than the prescribed activity. Because a nominal “100 mCi” prescription may be delivered as <100 mCi depending on radioactive decay and timing of administration, we prespecified >90 mCi (3.33 GBq) to define high-activity therapy when using administered dose data, corresponding operationally to the commonly prescribed 100 mCi dose. Prescribed activity was selected by the treating endocrinologist according to institutional practice and clinical features (eg, nodal burden, extrathyroidal extension, postoperative thyroglobulin). RAI activity was categorized as moderate (1.11-3.33 GBq [30-90 mCi]) or high (>3.33 GBq [>90 mCi]); doses <1.11 GBq (<30 mCi) were considered low activity and were excluded. Data on RAI preparation method (recombinant human TSH stimulation hormone [rhTSH] vs levothyroxine withdrawal) were available for a subset of patients and summarized descriptively.

### Exposures and covariates

The primary exposure was the administered activity of the first postoperative therapeutic RAI, categorized as moderate- or high-activity, as defined above. Exposure was fixed at the time of the initial postoperative RAI administration. Patients who received additional RAI during follow-up remained classified according to their initial activity, as subsequent RAI was considered a postbaseline event reflecting disease course rather than a censoring event. Covariates were abstracted from operative reports, final pathology reports, and structured fields within the institutional thyroid cancer database. Prespecified covariates included age at diagnosis, sex, primary tumor characteristics (size, focality, histologic variant), nodal characteristics (number, location, size of metastatic deposits, ENE, and American Joint Committee on Cancer [AJCC] N category), and additional pathologic features including mETE, angioinvasion, lymphatic invasion, and surgical margin status. Detailed variable definitions are provided in the Supplementary Methods [19].

### Outcomes

The primary outcome was time from diagnosis to first recurrence, defined as a transition from an excellent response to either biochemical or structural incomplete response. Response categories were adapted from the 2015 ATA response-to-therapy framework and operationalized using imaging findings and TSH-suppressed serum thyroglobulin/anti–thyroglobulin antibody trends. Structural recurrence was defined by radiographic or pathologic evidence of disease. Detailed outcome definitions and adjudication procedures are provided in the Supplementary Methods and Table S1 [19]. The secondary outcome was a composite of persistent or recurrent disease, defined as failure to achieve or maintain an excellent response at last follow-up. This composite outcome captured both patients who never achieved an excellent response (persistent disease) and those who initially achieved an excellent response but subsequently lost it (recurrent disease), reflecting the view, supported by contemporary guidelines and literature, that persistent and recurrent disease represent a continuum of post-therapeutic disease activity rather than discrete states [20-22].

### Follow-up period

Follow-up began at diagnosis to avoid defining cohort entry based on a postbaseline treatment event. For the primary outcome, patients were followed until the earliest of: (1) recurrence, (2) last available follow-up, or (3) death. For the composite outcome, an event was assigned if the patient did not have an excellent response at that final assessment and censoring otherwise. This approach accommodated variability in surveillance duration. Patients without any follow-up after diagnosis were excluded from the composite outcome analysis.

### Data handling

For covariates deemed essential to the exposure–outcome relationship, complete-case analysis was used. Variables with more than 50% missingness (eg, serum thyroglobulin) were excluded from multivariable and propensity-score analyses. The interval between thyroidectomy and the first RAI administration was calculated; when surgery date was unavailable (*n* = 138), it was imputed as 90 days after diagnosis [23]. Surgery dates were used only for descriptive timing analyses and were not required for cohort entry, exposure classification, or outcome ascertainment. Full details are provided in the Supplementary Methods [19].

### Statistical analysis

#### Baseline characteristics and covariate balance

Baseline characteristics were summarized for patients receiving moderate- or high-activity RAI. Covariate balance before and after inverse probability of treatment weighting (IPTW) was assessed using standardized mean differences (SMDs), with values <0.10 indicating acceptable balance. Weight stability was further summarized using the effective sample size. Stabilized weights were truncated at the 1st and 99th percentiles to improve numerical stability, with no observations excluded.

#### Primary analysis: IPTW-based treatment effect on recurrence

IPTW based on the propensity score was used to address confounding by indication, given that patients with more adverse clinicopathologic features were more likely to receive high-activity RAI and were also at increased risk of recurrence. By weighting individuals according to the inverse probability of receiving their observed treatment, IPTW generates a pseudopopulation in which measured baseline covariates are balanced across treatment groups. Propensity scores were estimated using prespecified covariates associated with both treatment selection and recurrence risk: age (per 5-year increase), tumor size, number of positive lymph nodes, size of the largest metastatic lymph node deposit, sex, mETE, and angioinvasion. Because recurrence is a relatively rare and specific outcome, a parsimonious propensity score model focusing on covariates most strongly related to treatment selection and recurrence risk was used to promote covariate overlap and stable weights.

#### Secondary analysis: IPTW-based treatment effect on the composite outcome

Using the same cohort and exposure groups, a separate IPTW analysis was conducted to evaluate the association between RAI activity and the composite outcome of persistent or recurrent disease. A distinct propensity score model incorporating a broader set of baseline clinicopathologic features was specified to better capture confounding by disease severity. All clinicopathologic variables summarized in Table 1 were included, except serum thyroglobulin (>50% missingness) and ENE. Although ENE is clinically relevant, it was rare and markedly imbalanced in this cohort, resulting in unstable propensity score estimation, and was therefore excluded. In both models (recurrence and composite outcomes), IPTW-weighted hazard ratios (HRs) and 95% confidence intervals (CIs) were estimated using marginal Cox proportional hazards models with robust standard errors. Kaplan–Meier and IPTW-weighted cumulative incidence curves were constructed for visualization. Sensitivity analyses included inverse probability of censoring weighting (IPCW) to account for potential informative censoring, as well as multivariable and doubly robust augmented IPTW Cox proportional hazards models to assess consistency of treatment effect estimates. Additional details are provided in the Supplementary Methods [19].

**Table 1:** Baseline characteristics of intermediate-risk patients with PTC by RAI activity group (moderate vs high), with standardized mean differences before and after inverse probability of treatment weighting (IPTW)

Characteristic		Intermediate patients with moderate or high activity RAI (*n* = 181)	Moderate activity *^a^* (*n* = 94)	High activity*^a^* (*n* = 87)	SMD before IPTW*^b^*	SMD after IPTW*^c^*
Age (years)*^d^*	Mean (SD); min–max	43.69 (15.14); 8-81	45.19 (14.23); 14-81	42.07 (16.00); 8-77	0.185	0.0480
Sex category, *n* (%)	Female	130 (71.8)	69 (73.4)	61 (70.1)	0.002	0.020
	Male	51 (28.3)	25 (26.6)	26 (30.2)		
Tumor size (cm)	Mean (SD); min–max	3.24 (2.02); 0.30-10.00	3.19 (1.91); 0.50-9.70	3.30 (2.14); 0.30-10.00	−0.003	−0.059
	Missing, *n* (%)	5 (2.8)	0 (0.0)	5 (5.7)		
Tumor focality, *n* (%)	Single	48 (26.5)	29 (30.9)	19 (21.8)	0.214	0.031
	Multifocal	128 (70.7)	64 (68.1)	64 (73.6)		
	Missing	5 (2.8)	1 (1.1)	4 (4.6)		
Number of positive lymph nodes	Mean (SD); min–max	3.71 (5.95); 0-40	2.13 (3.31); 0-20	5.44 (7.54); 0-40	−0.659	−0.134
	Missing, *n* (%)	1 (0.5)	0 (0)	1 (1.1)		
Lymph node location, *n* (%)	Central	140 (77.8)	76 (80.9)	64 (74.4)	0.216	0.018
	Lateral	41 (22.6)	18 (19.1)	23 (26.4)		
N Stage category, *n* (%)	N0	72 (39.8)	42 (44.7)	30 (34.5)	0.209	NA
	N1	109 (60.2)	52 (55.3)	57 (65.5)		
Microscopic extrathyroidal extension, *n* (%)	Yes	31 (17.1)	14 (14.9)	17 (19.5)	0.102	0.027
	No	150 (83.3)	80 (85.1)	70 (81.4)		
Angioinvasion, *n* (%)	Yes	30 (16.6)	15 (16.0)	15 (17.2)	0.102	0.045
	No	151 (83.9)	79 (84.0)	72 (83.7)		
lymphatic invasion, *n* (%)	Yes	26 (14.4)	16 (17.0)	10 (11.5)	0.206	0.088
	No	155 (86.1)	78 (83.0)	77 (89.5)		
Extranodal extension, *n* (%)	Yes	5 (2.8)	4 (4.3)	1 (1.1)	0.298	NA
	No	176 (97.8)	90 (95.7)	86 (100.0)		
Positive margins, *n* (%)	Yes	28 (15.5)	13 (13.8)	15 (17.2)	0.094	0.032
	No	153 (85.0)	81 (86.2)	72 (83.7)		
Size of largest metastatic lymph node deposit (cm)	Mean (SD); min–max	0.24 (0.58); 0-2.80	0.21 (0.50); 0-2.80	0.28 (0.65); 0-2.60	−0.179	−0.057
	Missing, *n* (%)	1 (0.5)	0 (0.0)	1 (1.1)		
Serum thyroglobulin (µg/L; ng/mL)*^e^*	Median (IQR); min–max	0.80 [0-3.79]; 0– 2417.00	0.23 [0-1.78]; 0-18.17	1.38 [0-8.09]; 0-2417.00	0.255	NA
	Missing *n* (%)	113 (62.4)	67 (71.3)	46 (52.9)		
Serum thyroglobulin antibody (IU/mL)*^e^*	Negative/Undetectable, *n* (%)	50 (27.6)	17 (18.1)	33 (37.9)	0.300	NA
	Positive/Detectable, *n* (%)	20 (11.0)	10 (10.6)	10 (11.5)		
	Missing, *n* (%)	111 (61.3)	67 (71.3)	44 (50.6)		
Histologic subtypes (Variant), *n* (%)	Classical	73 (40.3%)	44 (46.8%)	29 (33.3%)	0.310	0.005
	Follicular	53 (29.3)	32 (34.0)	21 (24.1)		
	Tall cell	14 (7.7)	6 (6.4)	8 (9.2)		
	Oncocytic	8 (4.4)	4 (4.3)	4 (4.6)		
	Other	4 (2.2)	1 (1.1)	3 (3.4)		
	Missing	29 (16.0)	7 (7.4)	22 (25.3)		

Abbreviations: IPTW, inverse probability of treatment weighting; NA, not applicable; RAI, radioactive iodine; SD, standard deviation; SMD, standardized mean difference.

^*a*^Counts and percentages reflect observed (unweighted) data.

^*b*^Standardized mean difference shown for moderate- vs high-activity groups only. Standardized mean difference ≥0.1 is considered clinically relevant.

^*c*^The standardized mean differences after inverse probability of treatment weighting using the composite-outcome (persistent or recurrent disease) propensity score model. The composite-outcome propensity score included the following prespecified clinicopathologic covariates: age (per 5-year increase), tumor size, number of positive lymph nodes, lymph node location (central vs lateral), tumor focality, microscopic extrathyroidal extension, angioinvasion, lymphatic invasion, positive margins, size of the largest metastatic lymph node deposit, and histologic variant. Serum thyroglobulin was excluded due to >50% missingness, and extranodal extension (ENE) was excluded due to severe imbalance (4/94 vs 1/87), which prevented stable estimation. Stabilized IPTW weights were truncated at the 1st and 99th percentiles.

^*d*^Minimum age reflects age at initial diagnosis; all patients were followed in an adult thyroid cancer clinic.

^*e*^Undetectable thyroglobulin imputed as 0. Serum thyroglobulin and thyroglobulin antibody measurements were obtained during follow-up and were available in a subset of patients. SMDs reflect complete-case comparisons only. Due to substantial missingness, these biomarkers were not included in propensity score models; therefore, SMD after IPTW is not applicable.

#### Multivariable prognostic cox model

In addition to the IPTW analyses, a multivariable Cox proportional hazards model was fit to identify prognostic factors associated with the composite endpoint, independent of RAI activity. Accordingly, RAI activity was not included as a covariate because the purpose of this model was not to estimate treatment effects. Covariates were selected a priori based on clinical relevance and included ENE, mETE, size of the largest metastatic lymph node deposit, age at diagnosis, angioinvasion, histologic variant, and lymph node location (central vs lateral). Additional variables were not included to avoid model overfitting.

All analyses were performed in R (version 4.4.1), with two-sided *P*-values <.05 considered statistically significant. Detailed statistical implementation and diagnostic procedures are provided in the Supplementary Methods [19].

## Results

**Figure 1:**
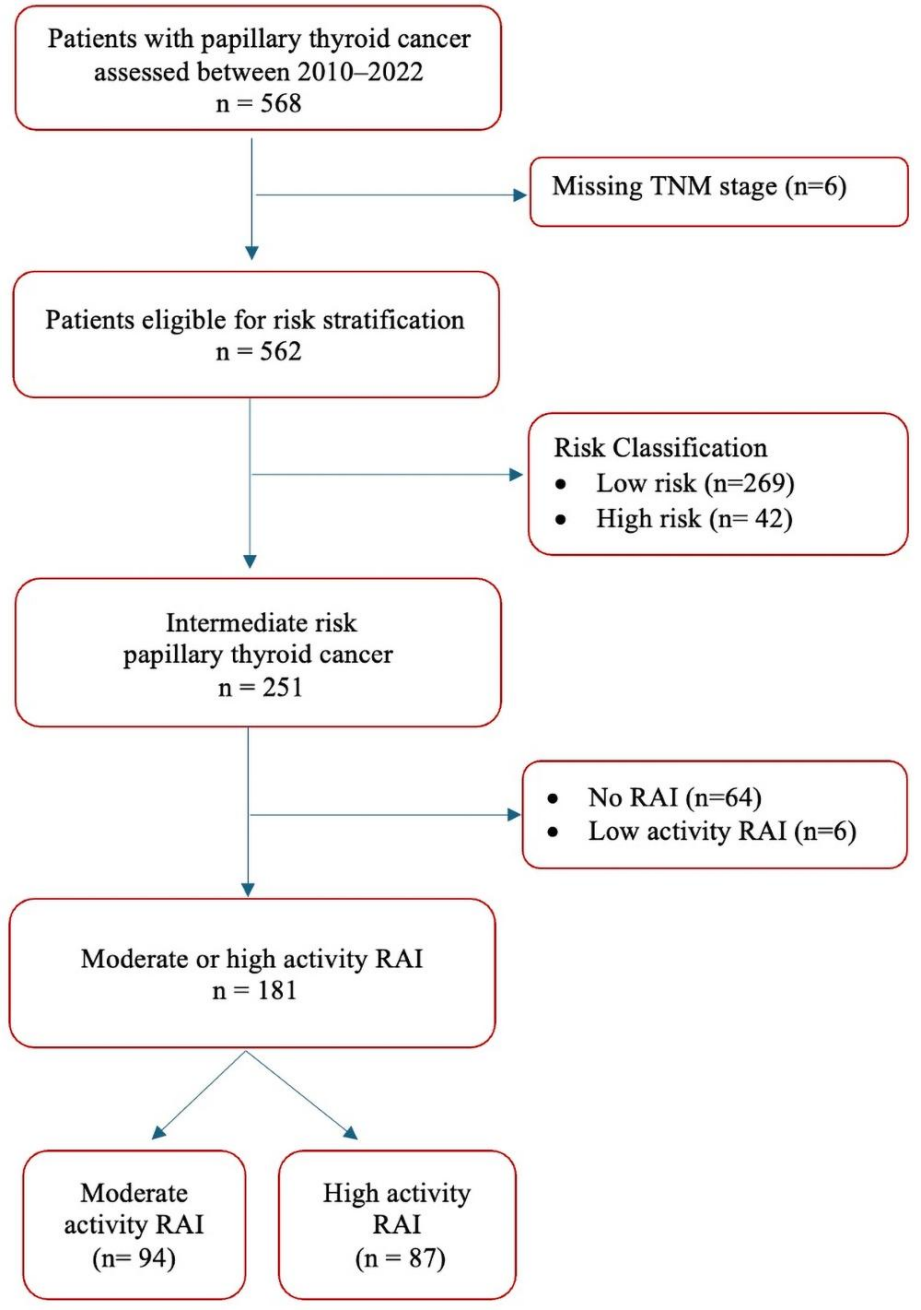
Strengthening the reporting of observational studies in epidemiology (STROBE) diagram of study cohort selection. Abbreviations: RAI, radioactive iodine; TNM, tumor–node–metastasis. Low activity: <30 mCi; moderate activity: 30-90 mCi; high activity >90 mCi.

Of 568 patients assessed, 6 were excluded due to unknown tumor–node–metastasis (TNM) stage. Among the remaining patients, 269 (48%) were classified as low risk, 42 (7%) as high risk, and 251 (45%) as intermediate risk. After excluding intermediate-risk patients who received no RAI (*n* = 64) or low-activity (*n* = 6), the final cohort included 181 patients treated with moderate- (*n* = 94) or high-activity (*n* = 87) RAI (Fig. 1). Median follow-up for the entire cohort was 51.08 months (IQR 12.29-83.05), with shorter follow-up in the moderate-activity group (30.3 vs 66.0 months). Completeness of baseline variables is summarized in Table 1. Information on the RAI preparation method was available for 25 of 181 patients (14%), most of whom underwent rhTSH stimulation (23/25, 92%). Preparation method distributions were similar across RAI activity groups (Table S2) [19]. The median interval between surgery and first RAI was 87.5 days (IQR 45-160) and was similar between activity groups (Fig. S1; Table S3) [19].

### Baseline characteristics

Among intermediate-risk patients who received moderate- or high-activity RAI (*n* = 181), the mean age was 43.8 years (SD 15.2), and 72% were female. The mean tumor size was 3.26 cm (SD 2.01), and patients had an average of 3.71 (SD 5.95) positive lymph nodes. Central lymph node involvement predominated (78%), compared to 23% with lateral involvement. Seventeen percent of patients had mETE, 17% had angioinvasion, 14% lymphatic invasion, 3% ENE, and 16% positive surgical margins. The mean size of the largest metastatic lymph node deposit was 0.24 cm (SD 0.58). Tumors were predominantly multifocal (71%), with classical histology most common (40%), and N1 disease was present in 60% of patients, more frequently among those receiving high-activity RAI.

Serum thyroglobulin was available in 70 patients (38.7%) more often in the high-activity group (43/87, 49.4%) than in the moderate-activity group (27/94, 28.7%). Among patients with available measurements, the median thyroglobulin concentration was 0.80 ng/mL (IQR 0-3.79), ranging from undetectable to 2417 ng/mL; thyroglobulin was undetectable in 23 patients (33%). All values were reported in µg/L (equivalent to ng/mL). Because serum thyroglobulin was missing in over half of patients and differed by treatment group, analyses involving thyroglobulin were descriptive and based on complete cases only, and thyroglobulin was not included in propensity score models or primary treatment-effect analyses. Anti-thyroglobulin antibodies were available in 70 patients (38.7%), demonstrated substantial missingness, and were therefore summarized descriptively only and excluded from propensity score models (Table 1).

At initial surgery, 138 patients (76.2%) underwent total thyroidectomy, 39 (21.5%) hemithyroidectomy, and 4 (2.2%) lobectomy. All patients with hemithyroidectomy or lobectomy subsequently underwent completion thyroidectomy before RAI. Central and lateral neck dissection were performed in 49 (27.1%) and 22 (12.2%) patients, respectively, while 110 (60.8%) had no documented neck dissection (Table S4) [19]. One patient (0.6%) died during follow-up in the high-activity group, without evidence of recurrence; this non–thyroid cancer death was treated as a censoring event.

Baseline covariate balance differed across analyses, reflecting the distinct propensity score models used. After IPTW, balance improved considerably in both analyses, with all SMDs <0.10 except lymph node count, which was markedly reduced. Stabilized weights were well behaved, with medians close to 1.00 and no extreme values after truncation. Propensity score distributions demonstrated adequate overlap between treatment groups, supporting the positivity assumption. Weight summaries, effective sample size, and additional IPTW diagnostics are provided in Tables S5-S9 and Fig. S2 [19].

### Administered RAI activity

**Figure 2:**
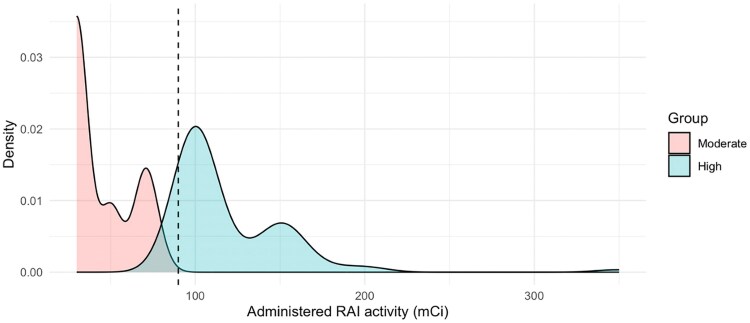
Distribution of administered radioactive iodine (RAI) activity by treatment group. Abbreviations: RAI, radioactive iodine. Density plot showing administered radioactive iodine activity (mCi) among intermediate-risk patients treated with moderate-activity (30-90 mCi) or high-activity (>90 mCi) radioactive iodine. The dashed vertical line marks the 90 mCi threshold used to define the treatment groups.

Among patients who received moderate-activity RAI (*n* = 94), the mean administered activity was 1.63 ± 0.67 GBq (44 ± 18 mCi), with a median of 1.11 GBq (IQR 1.11-2.52). In the high-activity group (*n* = 87), the mean administered activity was 4.41 ± 1.35 GBq (119 ± 36 mCi), with a median of 3.70 GBq (IQR 3.70-5.55). Administered activities were fully separated across the predefined dose categories (Fig. 2). Most patients (164/181, 91%) received a single RAI administration. Seventeen patients (9%) received additional RAI doses, all of which occurred after documentation of recurrence and represented retreatment rather than part of initial postoperative therapy (Table S10) [19].

### Primary outcome: recurrence

**Figure 3:**
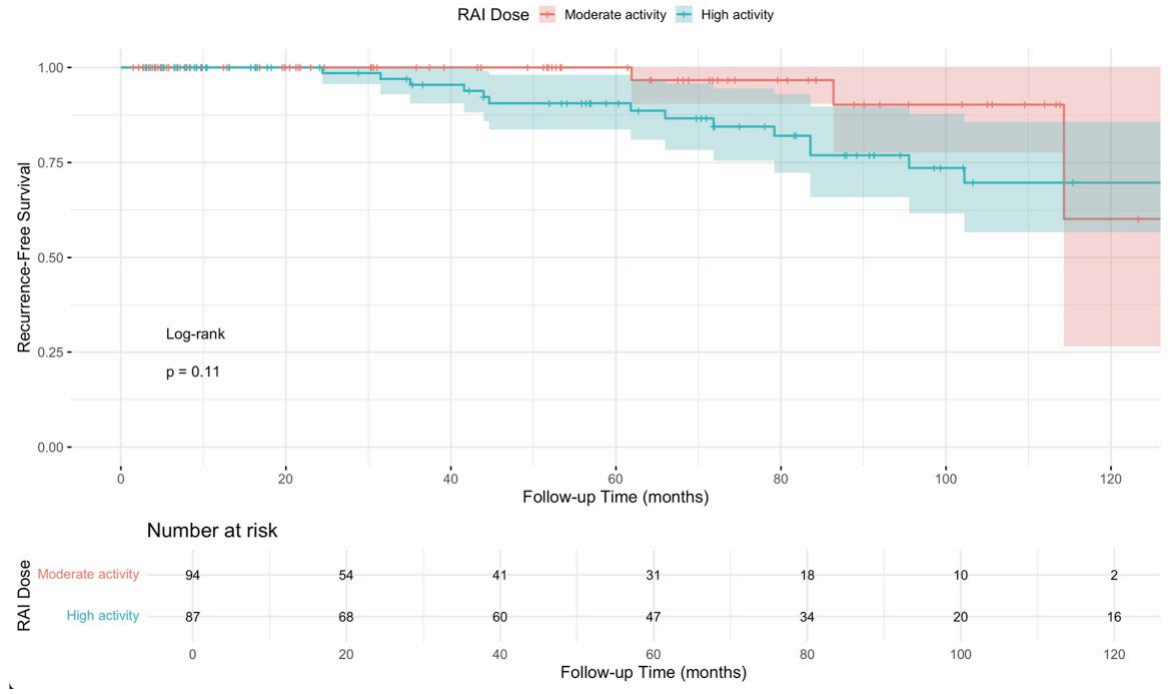
Kaplan–Meier curve for recurrence-free survival probability among patients with intermediate-risk papillary thyroid cancer treated with moderate- or high-activity radioactive iodine, stratified by activity: moderate (30-90 mCi), and high (>90 mCi). Abbreviations: RAI, radioactive iodine. Shaded bands represent the 95% confidence intervals around each survival curve. The log-rank test was used to compare survival distributions among groups. Censored data points are indicated by vertical marks.

Eighteen patients experienced recurrence: 3/94 (3.2%) in the moderate-activity group and 15/87 (17.2%) in the high-activity group (absolute difference, 14.0%). No recurrence events were recorded before initial RAI administration. Recurrence timing varied; 7/18 events (39%) occurred within 2 years of RAI administration, and the remaining 11 (61%) occurred later. In the moderate-activity group, 2 of 3 recurrences occurred within 2 years, whereas in the high-activity group, 5 of 15 recurrences occurred within 2 years and 10 occurred later. Of the 18 recurrence events, 16 were structural and 2 were biochemical. Structural recurrences were identified primarily through cross-sectional imaging, with detailed recurrence characteristics summarized in Table S11 [19]. Kaplan–Meier curves showed better recurrence-free survival in the moderate-activity group, but the difference was not statistically significant (log-rank *P* = .11; Fig. 3). In unadjusted and IPTW-weighted Cox models, high-activity RAI was not significantly associated with recurrence (HR 2.76; 95% CI, 0.79-9.64; *P* = .11 and HR 2.73; 95% CI, 0.74-10.01; *P* = .13, respectively) (Table 2).

**Table 2:** Unadjusted and inverse probability of treatment weighted hazard ratios comparing high- vs moderate-activity radioactive iodine in intermediate-risk papillary thyroid cancer

Outcome	Model	Events (moderate, *n* = 94)	Events (high, *n* = 87)	Hazard ratio (95% CI)*^a^*	*P*-value
Recurrence only	Unadjusted	3	15	2.76 (0.79-9.64)	.11
	IPTW-Weighted HR (ATE)			2.73 (0.74-10.01)	.13
Persistent or recurrent disease (composite outcome)	Unadjusted	22 (21 persistent, 1 recurrent)	39 (35 persistent, 4 recurrent)	1.04 (0.60-1.79)	.90
	IPTW-Weighted (ATE)			0.97 (0.53-1.76)	.90

RAI activity groups: moderate-activity (30-90 mCi); High-activity (>90 mCi).

Abbreviations: ATE, average treatment effect; CI, confidence interval; HR, hazard ratio; IPTW, inverse probability of treatment weighting.

^*a*^Moderate-activity RAI (30-90 mCi) used as the reference group.

### Persistent or recurrent disease composite outcome

**Figure 4:**
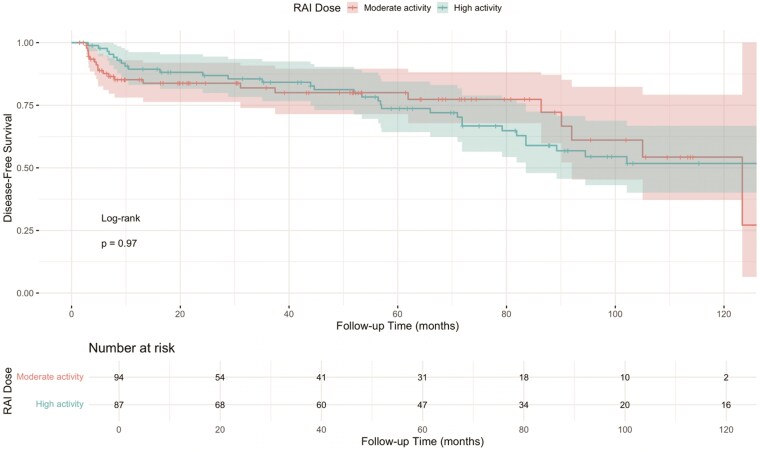
Kaplan–Meier curves for survival free from persistent or recurrent disease (composite endpoint) among patients with intermediate-risk papillary thyroid cancer treated with moderate- or high-activity radioactive iodine, stratified by administered activity: moderate (30-90 mCi), and high (>90 mCi). Abbreviations: RAI, radioactive iodine. Shaded bands represent the 95% confidence intervals around each survival curve. The log-rank test was used to compare survival distributions among groups. Censored data points are indicated by vertical marks.

At the last available follow-up, 117 of 181 patients (65%) were disease-free, while 61 (34%) met the composite endpoint of persistent or recurrent disease. Of these 61 patients, 5 had a documented recurrence and 56 had persistent disease (Table 2). The composite endpoint occurred in 22/94 (23.4%) patients in the moderate-activity group and 39/87 (44.8%) in the high-activity group (absolute difference, 21.4%). No significant association with RAI activity was found in either unadjusted (HR 1.04; 95% CI, 0.60-1.79; *P* = .90) or IPTW-adjusted models (HR 0.97; 95% CI, 0.53-1.76; *P* = .90) (Table 2). Kaplan–Meier curves also demonstrated no difference in disease-free survival between groups (log-rank *P* = .97) (Fig. 4). IPTW-weighted cumulative incidence curves for both outcomes are presented in Fig. S3 [19]. Sensitivity analyses accounting for differential follow-up using IPCW, multivariable Cox regression adjusting directly for baseline covariates, and doubly robust augmented IPTW Cox models yielded results consistent with the primary IPTW analyses (Tables S12-S16) [19].

### Factors associated with persistent or recurrent disease

**Figure 5:**
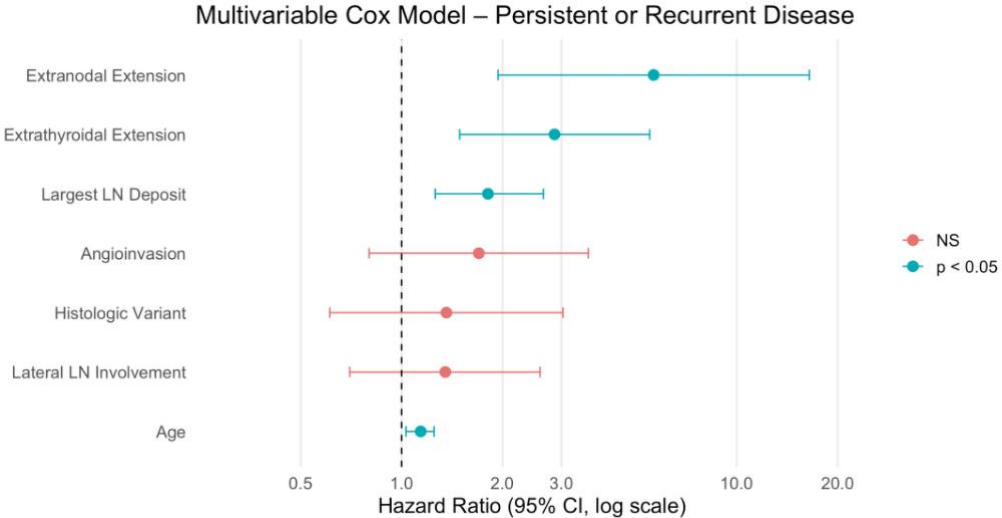
Forest plot of adjusted hazard ratios for the association between clinicopathologic characteristics and persistent or recurrent disease in intermediate-risk papillary thyroid cancer. Abbreviations: CI, confidence interval; LN, lymph node; NS, not significant .

In the multivariable model, persistent or recurrent disease was associated with ENE (HR 5.65; 95% CI, 1.94-16.46; *P* = .002), mETE (HR 2.86; 95% CI, 1.49-5.50; *P* = .001), larger metastatic lymph node deposit size (per 1-cm increase: HR 1.81; 95% CI, 1.26-2.65; *P* = .001), and older age (per 5-year increase: HR 1.14; 95% CI, 1.03-1.25; *P* = .008). Angioinvasion, high-risk histologic subtype, and lateral lymph node involvement were not statistically associated with persistent or recurrent disease (Table 3; Fig. 5). Model diagnostics are reported in the Supplementary Materials (Tables S17 and S18; Figs. S4-S7) [19].

**Table 3:** Adjusted hazard ratios for the association between clinicopathologic characteristics and persistent or recurrent disease in intermediate-risk papillary thyroid cancer

Characteristic	Categories or units	HR (95% CI)	*P*-value
Extranodal extension	No (ref)/Yes	5.65 (1.94-16.46)	.002
Extrathyroidal extension	No (ref)/Yes	2.86 (1.49-5.50)	.001
Size of largest metastatic deposit	Per 1 cm increase	1.81 (1.26-2.65)	.001
Age	Per 5-year increase	1.14 (1.03-1.25)	.008
Angioinvasion	No (ref)/yes	1.70 (0.80-3.61)	.17
Histologic variant	Classical (ref)/high-risk subtype	1.36 (0.61-3.03)	.46
Lateral LN Involvement	Central (ref)/lateral	1.35 (0.70-2.59)	.37

Abbreviations: CI, confidence interval; HR, hazard ratio.

## Discussion

In this retrospective cohort of adults with intermediate-risk PTC, we found no evidence that high-activity RAI improved recurrence-free survival compared with moderate-activity RAI. Recurrence rates were low overall, and findings were consistent when disease burden was assessed using a broader composite endpoint of persistent or recurrent disease. Together, these results suggest that higher RAI activity does not confer a clear oncologic advantage over moderate activity in this population.

Prior studies evaluating RAI activity in intermediate-risk PTC have yielded inconsistent results, likely reflecting heterogeneity in risk definitions, dose thresholds, and outcome measures [15, 16, 24]. Randomized trials in low-risk disease have demonstrated comparable efficacy between low- and high-activity RAI, whereas evidence in intermediate-risk populations remains limited and less consistent [25, 26]. Several observational studies report similar outcomes between lower and higher RAI activities in selected intermediate- or high-risk groups, including patients with N1 disease, while others suggest potential benefit of higher activity in subgroups with greater nodal burden or elevated stimulated thyroglobulin [8, 27-31].

Our study is among the first to evaluate moderate-activity RAI (1.11-3.33 GBq [30-90 mCi]) as a distinct reference category in intermediate-risk PTC. By limiting the cohort to intermediate-risk patients and applying IPTW to account for treatment selection bias, we aimed to provide clinically relevant, real-world evidence on the comparative effectiveness of RAI activity. Using recurrence-free survival as the primary outcome also enhances clinical interpretability over short-term biochemical markers.

We report that the presence of ENE, mETE, larger metastatic lymph node size, and increasing age were independently associated with a higher risk of persistent or recurrent disease. ENE was strongly associated with persistent or recurrent disease, reinforcing prior evidence that its presence, even with limited nodal burden, provides meaningful prognostic information [9, 32-34]. This aligns with findings by Kim et al (2019), who demonstrated higher recurrence risk among patients with ENE despite low-volume nodal disease [33]. Together, these findings support consideration of ENE as an independent risk modifier in clinical decision-making.

Although patients with gross ETE were excluded, microscopic ETE predicted adverse outcomes. This contrasts with studies such as ITCO#4 (Forleo et al, 2021) and Castagna et al (2018), which found limited prognostic impact of mETE in small tumors [35, 36]. However, our findings align with those of Parvathareddy et al (2021) and Marongiu et al (2024), who reported worse disease-free survival with mETE [37, 38]. These discrepancies likely reflect interobserver variability in pathology interpretation and a lack of standardized diagnostic criteria. Larger, multicenter studies with consistent pathology review are needed to clarify its relevance. In the interim, management decisions should be individualized, with closer follow-up for mETE primarily when accompanied by additional adverse features.

We also found that larger metastatic lymph node deposits and increasing age were independently associated with higher risk, supporting their value as continuous predictors beyond traditional nodal counts and AJCC staging [18, 39, 40]. Although clinicopathologic features are essential to risk stratification, their predictive accuracy is limited in isolation. Future risk models may incorporate dynamic biochemical markers (eg, serial thyroglobulin) and molecular data (eg, TERT mutations) to better personalize treatment [41-44].

Refining risk stratification within intermediate-risk PTC could help identify patients who may benefit from higher RAI doses and intensive monitoring, while allowing others to safely receive moderate doses. Rather than uniform dose escalation, treatment decisions should be guided by adverse features such as nodal disease burden, ENE, and mETE, alongside dynamic risk assessment during follow-up [18, 45]. This risk-adapted approach may optimize oncologic outcomes while minimizing unnecessary treatment-related toxicity.

Although our study focuses on RAI activity among treated patients rather than RAI omission, the lack of recurrence benefit with higher activity has important implications for risk-adapted care. If escalation beyond moderate activity does not improve outcomes in intermediate-risk disease, the necessity and intensity of postoperative RAI in selected patients warrant reconsideration. Ongoing randomized trials, including the French INTERMEDIATE study (NCT04290663), will clarify whether some intermediate-risk patients may safely avoid RAI altogether [46]. Our findings contribute to this evolving paradigm by demonstrating that, among patients who receive RAI, higher activity does not confer additional oncologic benefit.

This study has several strengths. Notably, the moderate-activity range examined reflects routine practice in North American centers, but has been rarely evaluated as a distinct comparator. By focusing on a well-defined intermediate-risk cohort and applying IPTW, we provide real-world evidence with improved covariate balance compared with standard regression approaches. The use of recurrence-free survival as the primary outcome enhances clinical relevance, and a median follow-up of just over 4 years allowed sufficient time to observe recurrences. Results were consistent in sensitivity analyses accounting for informative censoring using IPCW. Finally, our analysis of clinicopathologic factors associated with persistent or recurrent disease offers hypothesis-generating insights that may guide future research and help refine management strategies for patients at elevated risk.

### Limitations

First, the small number of recurrence events limited statistical power and resulted in imprecise effect estimates with wide CIs. With only 18 events, this study was underpowered to detect anything other than large treatment effects. As a result, the CIs include clinically meaningful benefit or harm, and the absence of statistical significance should not be interpreted as evidence of equivalence. The higher point estimate observed in the high-activity group should therefore not be interpreted as evidence of harm and is more plausibly attributable to residual confounding, imperfect covariate balance, and differential follow-up. To address outcome sparsity, we performed a secondary analysis using a composite endpoint of persistent or recurrent disease, which occurred more frequently, and yielded effect estimates closer to null. Persistent disease occurred at similar proportions in both groups, indicating that the composite outcome did not obscure a meaningful difference in recurrence risk. Second, confounding by indication is possible. Patients receiving high-activity RAI may have had more aggressive disease not fully captured by available variables. Although IPTW accounted for measured clinicopathologic confounders, residual bias may remain. In particular, ENE could not be incorporated into the propensity score model, and postoperative thyroglobulin and molecular marker data were incompletely or not available. Residual confounding related to these factors, therefore, cannot be excluded. Third, follow-up duration differed between RAI activity groups, with shorter follow-up in the moderate-activity group. This likely reflects earlier practice patterns favoring higher RAI activity, and more prolonged surveillance among patients perceived to be at higher risk who received high-activity RAI. To address the possibility of informative censoring arising from differential follow-up, we performed sensitivity analyses using IPCW, which yielded results consistent with the primary analyses. Residual bias from unmeasured predictors of censoring cannot be excluded. Fourth, ATA risk classification was applied algorithmically based on tumor and nodal characteristics, rather than clinician-assigned categories [18]. Although this ensured consistency across the cohort, it may have introduced misclassification, particularly for features that were undocumented or interpreted variably in clinical practice. Finally, the study spanned a period of evolving practice (2010-2022), during which guideline changes promoted more selective RAI use. Although we adjusted for key clinical variables, unmeasured provider- or institutional-level factors may have influenced treatment and follow-up, and generalizability to centers with different practice patterns may be limited. Accordingly, these findings should be interpreted as hypothesis-generating and require confirmation in larger prospective or randomized studies before being applied to routine clinical practice.

### Conclusion

These findings support a risk-adapted approach to RAI activity in intermediate-risk PTC. In this cohort, high-activity RAI did not improve recurrence or disease control compared with moderate-activity RAI, and clinicopathologic features such as nodal burden and mETE were associated with persistent or recurrent disease. Together, these results suggest that escalation beyond moderate-activity RAI is unlikely to provide additional oncologic benefit for most intermediate-risk patients and should not be routine. Future noninferiority studies are needed to confirm whether moderate-activity RAI can safely replace higher activities and to identify subgroups who may benefit from further de-escalation or omission of RAI.

## Data Availability

Restrictions apply to the availability of some or all data generated or analyzed during this study to preserve patient confidentiality or because they were used under license. The corresponding author will on request detail the restrictions and any conditions under which access to some data may be provided.

## References

[1] Lim H, Devesa SS, Sosa JA, Check D, Kitahara CM. Trends in thyroid cancer incidence and mortality in the United States, 1974-2013. JAMA. 2017;317(13):1338‐1348. 28362912 10.1001/jama.2017.2719 PMC8216772

[2] Hellman P, Norlén O, Stålberg P, Daskalakis K. Thyroid cancer. In: Yalcin S, Öberg K, eds. Neuroendocrine Tumours: Diagnosis and Management. Springer; 2024:445‐483.

[3] Canadian Cancer Society . Canadian Cancer Statistics 2023. Accessed November 2023. https://cancer.ca/Canadian-Cancer-Statistics-2023-EN

[4] Ellison L . Progress in net cancer survival in Canada over 20 years. Health Rep. 2018;29(9):10‐18. 30230521

[5] Chen Q, Zou X, Liu F, et al Restratification of intermediate risk factors on the recurrence of papillary thyroid carcinoma: a retrospective cohort study. Int J Surg. 2025;111(1):884‐890. 38976907 10.1097/JS9.0000000000001945 PMC11745661

[6] Grogan RH, Kaplan SP, Cao H, et al A study of recurrence and death from papillary thyroid cancer with 27 years of median follow-up. Surgery. 2013;154(6):1436‐1447. 24075674 10.1016/j.surg.2013.07.008

[7] Llamas-Olier AE, Cuéllar DI, Buitrago G. Intermediate-risk papillary thyroid cancer: risk factors for early recurrence in patients with excellent response to initial therapy. Thyroid. 2018;28(10):1311‐1317. 30105948 10.1089/thy.2017.0578

[8] Chandekar KR, Satapathy S, Bal C. Impact of radioiodine therapy on recurrence and survival outcomes in intermediate-risk papillary thyroid carcinoma: a systematic review and meta-analysis. Clin Endocrinol (Oxf). 2024;100(2):181‐191. 38050454 10.1111/cen.15001

[9] Suh S, Pak K, Seok JW, Kim IJ. Prognostic value of extranodal extension in thyroid cancer: a meta-analysis. Yonsei Med J. 2016;57(6):1324‐1328. 27593858 10.3349/ymj.2016.57.6.1324 PMC5011262

[10] Zhao M, Shi X, Zhang J, et al Radioactive iodine ablation can reduce the structural recurrence rate of intermediate-risk papillary thyroid microcarcinoma: a meta-analysis. Comput Math Methods Med. 2022;2022:8028846. 36110571 10.1155/2022/8028846 PMC9470344

[11] Klain M, Nappi C, Zampella E, et al Ablation rate after radioactive iodine therapy in patients with differentiated thyroid cancer at intermediate or high risk of recurrence: a systematic review and meta-analysis. Eur J Nucl Med Mol Imaging. 2021;48(13):4437‐4444. 34142215 10.1007/s00259-021-05440-x PMC8566414

[12] Vardarli I, Weidemann F, Aboukoura M, Herrmann K, Binse I, Görges R. Longer-term recurrence rate after low versus high dose radioiodine ablation for differentiated thyroid cancer in low- and intermediate-risk patients: a meta-analysis. BMC Cancer. 2020;20(1):1‐9. 10.1186/s12885-020-07029-3 PMC7296693 32539683

[13] Aghaei A, Ayati N, Shafiei S, Abbasi B, Zakavi S. Comparison of treatment efficacy 1 and 2 years after thyroid remnant ablation with 1110 versus 5550 MBq of iodine-131 in patients with intermediate-risk differentiated thyroid cancer. Nucl Med Commun. 2017;38(11):927. 28834790 10.1097/MNM.0000000000000730

[14] Sehdev S, Gotfrit J, Elias M, Stein BD. Impact of systemic delays for patient access to oncology drugs on clinical, economic, and quality-of-life outcomes in Canada: a call to action. Curr Oncol. 2024;31(3):1460‐1469. 38534943 10.3390/curroncol31030110 PMC10969399

[15] Gao H, Huang J, Dai Q, Su J. Radioiodine (131I) treatment decision-making for low- and intermediate-risk differentiated thyroid cancer. Arch Endocrinol Metab. 2023;67(2):197‐205. 36651706 10.20945/2359-3997000000538 PMC10689029

[16] Chen P, Luo J, Ouyang W, et al Clinical outcomes of low-dose and high-dose postoperative radioiodinetherapy in intermediate-risk papillary thyroid carcinoma patients with low postoperative stimulated thyroglobulin (1–20ng/mL) and non-structural or functional metastasis: study protocol for a randomized controlled trial. 2021. doi:10.21203/RS.3.RS-59344/V1

[17] Onoda N, Ito Y, Miya A, Kihara M, Miyauchi A. Predictors of distant metastatic recurrence in intermediate-risk papillary thyroid carcinoma. World J Surg. 2025;49(1):117‐123. 39187905 10.1002/wjs.12289 PMC11711113

[18] Haugen BR, Alexander EK, Bible KC, et al 2015 American Thyroid Association management guidelines for adult patients with thyroid nodules and differentiated thyroid cancer. Thyroid. 2016;26(1):1‐133. 26462967 10.1089/thy.2015.0020 PMC4739132

[19] Jay M, Lega IC, Villemure-Poliquin N, Goens C, Zahedi A. 2026. Supplementary materials for “Moderate- versus high-activity radioactive iodine in intermediate-risk papillary thyroid cancer: a Canadian cohort study”. Figshare. Accessed March 13, 2026. doi:10.6084/m9.figshare.31690879. PMC13249021 42272564

[20] Scheffel RS, Zanella AB, Dora JM, Maia AL. Timing of radioactive iodine administration does not influence outcomes in patients with differentiated thyroid carcinoma. Thyroid. 2016;26(11):1623‐1629. 27549175 10.1089/thy.2016.0038

[21] Ringel MD, Sosa JA, Baloch Z, et al 2025 American Thyroid Association management guidelines for adult patients with differentiated thyroid cancer. Thyroid. 2025;35(8):841‐985. 40844370 10.1177/10507256251363120 PMC13090833

[22] Al-Haideri MH, Othman M, Ahmad D, Alshamsi M, Al Janabi M. Incidence of persistence and recurrence of differentiated thyroid cancer in post-surgical cases from a Tertiary Care Hospital in Dubai, United Arab Emirates. Cureus. 2024;16(7):e63555. 39087148 10.7759/cureus.63555 PMC11289654

[23] Coca-Pelaz A, Rodrigo JP, Shah JP, et al Recurrent differentiated thyroid cancer: the current treatment options. Cancers (Basel). 2023;15(10):2692. 37345029 10.3390/cancers15102692 PMC10216352

[24] Zhao H, Gong Y. Radioactive iodine in low- to intermediate-risk papillary thyroid cancer. Front Endocrinol (Lausanne). 2022;13:960682. 36034423 10.3389/fendo.2022.960682 PMC9402902

[25] Mirghani H, Altidlawi MI, Altedlawi Albalawi IA. The optimal activity of radioactive iodine for remnant ablation in low/intermediate risk differentiated thyroid carcinoma: a continuous controversy and meta-analysis. Cureus. 2021;13:e12937. 33643743 10.7759/cureus.12937 PMC7885745

[26] Castagna M, Cevenini G, Theodoropoulou A, et al Post-surgical thyroid ablation with low or high radioiodine activities results in similar outcomes in intermediate risk differentiated thyroid cancer patients. Eur J Endocrinol. 2013;169(1):23‐29. 23594687 10.1530/EJE-12-0954

[27] Li X, Zheng H, Ma C, et al Higher adjuvant radioactive iodine therapy dosage helps intermediate-risk papillary thyroid carcinoma patients achieve better therapeutic effect. Front Endocrinol (Lausanne). 2024;14:1307325. 38298190 10.3389/fendo.2023.1307325 PMC10829775

[28] Jin M, Ahn J, Lee YM, et al Clinical outcomes of N1b papillary thyroid cancer patients treated with two different doses of radioiodine ablation therapy. Endocrinol Metab. 2020;35(3):602‐609. 10.3803/EnM.2020.741 PMC7520585 32981302

[29] Odil EE, Ward KR, Davis RT, et al Radioactive iodine therapy dose impact on recurrence and survival in N1 papillary thyroid cancer. Nucl Med Commun. 2025;46(2):113‐119. 39604284 10.1097/MNM.0000000000001936

[30] Seo M, Kim YS, Lee JC, et al Low-dose radioactive iodine ablation is sufficient in patients with small papillary thyroid cancer having minor extrathyroidal extension and central lymph node metastasis (T3 N1a). Clin Nucl Med. 2017;42(11):842‐846. 28832376 10.1097/RLU.0000000000001812

[31] Zhang Y, Wang C, Zhang X, Li H, Li X, Lin Y. 30mCi radioactive iodine achieving comparative excellent response in intermediate/high-risk nonmetastatic papillary thyroid cancer: a propensity score matching study. Endocrine. 2018;62(3):655‐662. 30145748 10.1007/s12020-018-1724-z

[32] Li G, Pan Z, Tao W, et al Prognostic implications of extranodal extension in papillary thyroid carcinomas: a propensity score matching analysis and proposal for incorporation into current tumor, lymph node, metastasis staging. Surgery. 2021;171:368‐376. 34482990 10.1016/j.surg.2021.07.018

[33] Kim HI, Hyeon J, Park S, et al Impact of extranodal extension on risk stratification in papillary thyroid carcinoma. Thyroid. 2019;29(7):963‐970. 31025609 10.1089/thy.2018.0541 PMC6648218

[34] Zhou TH, Lin B, Wu F, et al Extranodal extension is an independent prognostic factor in papillary thyroid cancer: a propensity score matching analysis. Front Endocrinol (Lausanne). 2021;12:759049. 34803921 10.3389/fendo.2021.759049 PMC8595930

[35] Forleo R, Grani G, Alfò M, et al Minimal extrathyroidal extension in predicting 1-year outcomes: a longitudinal multicenter study of low- to intermediate-risk papillary thyroid carcinoma (ITCO#4). Thyroid. 2021;31(12):1814‐1821. 34541894 10.1089/thy.2021.0248

[36] Castagna M, Forleo R, Maino F, et al Small papillary thyroid carcinoma with minimal extrathyroidal extension should be managed as ATA low-risk tumor. J Endocrinol Invest. 2018;41(9):1029‐1035. 29470826 10.1007/s40618-018-0854-8

[37] Parvathareddy SK, Siraj AK, Qadri Z, et al Microscopic extrathyroidal extension results in increased rate of tumor recurrence and is an independent predictor of patient outcome in Middle Eastern papillary thyroid carcinoma. Front Oncol. 2021;11:724432. 34926245 10.3389/fonc.2021.724432 PMC8671701

[38] Marongiu A, Nuvoli S, De Vito A, Vargiu S, Spanu A, Madeddu G. Minimal extrathyroid extension as the only risk factor in patients with papillary thyroid carcinoma: its clinical impact on recurrence and outcome during long-term follow-up. Biomedicines. 2024;12(2):350. 38397952 10.3390/biomedicines12020350 PMC10886778

[39] Ito Y, Miyauchi A, Fujishima M, et al Prognostic significance of patient age in papillary thyroid carcinoma with no high-risk features. Endocr J. 2022;69(9):1131‐1136. 35431281 10.1507/endocrj.EJ22-0056

[40] Ywata de Carvalho A, Kohler HF, Gomes CC, Vartanian JG, Kowalski LP. Predictive factors for recurrence of papillary thyroid carcinoma: analysis of 4,085 patients. Acta Otorhinolaryngol Ital. 2021;41(3):236. 34264917 10.14639/0392-100X-N1412 PMC8283398

[41] Cho JS, Kim HK. Thyroglobulin levels as a predictor of papillary thyroid cancer recurrence after thyroid lobectomy. Anticancer Res. 2022;42(11):5619‐5627. 36288865 10.21873/anticanres.16070

[42] Li X, Tian T, Shi K, Sun C, Huang R. Impact of thyroglobulin changes on clinical outcomes of differentiated thyroid cancer with biochemical incomplete response. Endocrine. 2025;89:1‐10. 40346325 10.1007/s12020-025-04247-2

[43] Huang J, Wang J, Xv J, Wang J, Wang G, Zhao Y. Genetic alterations and allele frequency of BRAF V600E and TERT mutation in papillary thyroid carcinoma with intermediate-to-high recurrence risk: a retrospective study. Clin Exp Med. 2024;24(1):76. 38607456 10.1007/s10238-024-01320-4 PMC11014806

[44] Sang Y, Hu G, Xue J, Chen M, Hong S, Liu R. Risk stratification by combining common genetic mutations and TERT promoter methylation in papillary thyroid cancer. Endocrine. 2024;85(1):304‐312. 38356100 10.1007/s12020-024-03722-6

[45] van Velsen EFS, Verburg FA. Adjuvant radioiodine for intermediate-risk papillary thyroid cancer—to treat or not to treat. J Clin Endocrinol Metab. 2023;108(10):e1149‐e1150. 36964916 10.1210/clinem/dgad171 PMC10505540

[46] Malandrino P, Tumino D, Russo M, et al Consider or not consider: the unsolved question on the use of radioactive iodine for differentiated thyroid cancer with low- to intermediate-risk of recurrence. Endocrine. 2025;87(2):658‐666. 39322929 10.1007/s12020-024-04027-4 PMC11811245

